# Lumbo-sacral motion conserved after isthmic reconstruction: long-term results

**DOI:** 10.1007/s11832-014-0560-9

**Published:** 2014-01-29

**Authors:** C. de Bodman, F. Bergerault, B. de Courtivron, C. Bonnard

**Affiliations:** CHU Clocheville, 49 Boulevard Béranger, 37044 Tours Cedex, France

**Keywords:** Isthmic reconstruction, Spondylolisthesis, Lumbo-sacral motion

## Abstract

**Purpose:**

The purpose of this study was to analyze the clinical and radiological results of repair of the interarticularis pars defect by a modified Buck’s repair technique in patients with symptomatic spondylolysis with grade 1 spondylolisthesis.

**Summary of background data:**

These patients with painful spondylolisthesis are the most eligible for direct repair of the defect rather than lumbo-sacral fusion in an attempt to save motion segments.

**Methods:**

Forty-six patients with symptomatic spondylolysis with grade 1 spondylolisthesis and normal L4–L5 and L5–S1 disks, following the failure of conservative treatment, underwent surgery between 1988 and 2010. All interventions involved direct pars repair by a modified Buck’s repair technique with internal fixation of the defect using screws and cancellous bone grafting. The Oswestry Disability Index (ODI) was used to evaluate the functional outcome. Healing of the pars defect was assessed by plain radiographs and computed tomography (CT) scanning. Motion of the L4–L5 and L5–S1 segments was measured with dynamic radiographs in flexion and extension.

**Results:**

Thirty-five patients were evaluated. The mean follow-up period was 10 years. Functional outcome was excellent in 22 patients (ODI ≤ 10) and good for 8 patients (10 < ODI ≤ 20); five patients continued to have pain (ODI >20). Isthmus bone union occurred in 32 of 35 patients (91.4 %). L4–L5 motion was conserved with a mean angle of 11.8° (0–22); the mean lumbo-sacral angle was 9.9° (0–21).

**Conclusion:**

Direct repair of spondylolisthesis was described to avoid fusion in young patients with slight slipping and painful symptoms. A modified Buck’s repair technique allows the conservation of L4–L5 motion with a rate of consolidation comparable to other series. The anatomy and stability of the spine were normalized by restoring the continuity of the loose posterior elements using this modified Buck’s technique.

## Introduction

Direct repair of spondylolisthesis was described by Buck [1] to avoid fusion in young patients with slight slipping and painful symptoms resistant to conservative treatment. Conservative measures include physical therapy and bracing with a thoraco-lumbar and sacral orthosis. This approach is successful in most patients [2]. Klein et al. [3] described successful clinical outcome in 84 % of cases after 1 year.

Those children with slight slipping who remained symptomatic require surgical treatment [4, 5]. Two procedures are proposed: posterior fusion or isthmic repair. Posterior fusion of the affected level has been widely used in these patients, with good results in 75–100 % of cases [6]. However, surgical fusion of the lumbar spine results in a loss of motion at the fused site, potentially increasing the loading on adjacent segments. Symptomatic degeneration warranting additional surgery occurred in 16.5 % of patients at 5 years and 36.1 % at 10 years follow-up [7] of one series of 215 patients who underwent surgical fusion.

Isthmic repair is also indicated. Conditions for good results with isthmic repair were proposed by Buck [1]: the gap in the neural arch was less than 3 or 4 mm and without spina occulta. With isthmic repair, the continuity of the loose posterior elements is restored to normalize the anatomy and stability of the spine. The technique avoided fusion, thereby preserving movement [8].

The aims of this study were to both evaluate the rate of consolidation and analyze the long-term L4–L5 and L5–S1 motion in patients undergoing a modified Buck’s surgical repair technique with.

## Materials and methods

The study was a retrospective analysis of 46 patients (22 female; 24 male) operated on between 1988 and 2010 for symptomatic lumbar spondylolisthesis. The mean age at the time of operation was 13.7 ± 2.7 years (range 7–19). Conservative treatment had failed in all cases with persistent pain. Surgery was indicated after a year of conservative treatment without success. The conservative treatment consisted of rest, physiotherapy, and brace immobilization. All sports were contraindicated during the painful period. Brace immobilization was done for 6 weeks if it was an acute pain, started less than 3 weeks previously. The treatment goal was to achieve consolidation.

Patients with slip percentages higher than 30 % or significant disk degeneration were not included because posterior fusion was done.

All patients underwent neurological examination conducted by the same independent physician. At revision, the subjective outcome was assessed using the Oswestry Disability Index (ODI) [9]. The ODI was not measured before surgery. The ODI evaluates subjective low-back disability. According to Fairbank et al. [10], the index evaluates the degree of low-back disability and is scored as follows: 0–19 = minimal disability; 20–39 = moderate disability; 40–59 = severe disability; and ≥60 = crippled.

### Radiological assessment

For the last 5 years, magnetic resonance imaging (MRI) was done before each surgery to analyze the state of the disk. Before this date, access to MRI was limited and more expensive.

Consolidation was evaluated with computed tomography (CT) 2 months after surgery.

**Fig. 1:**
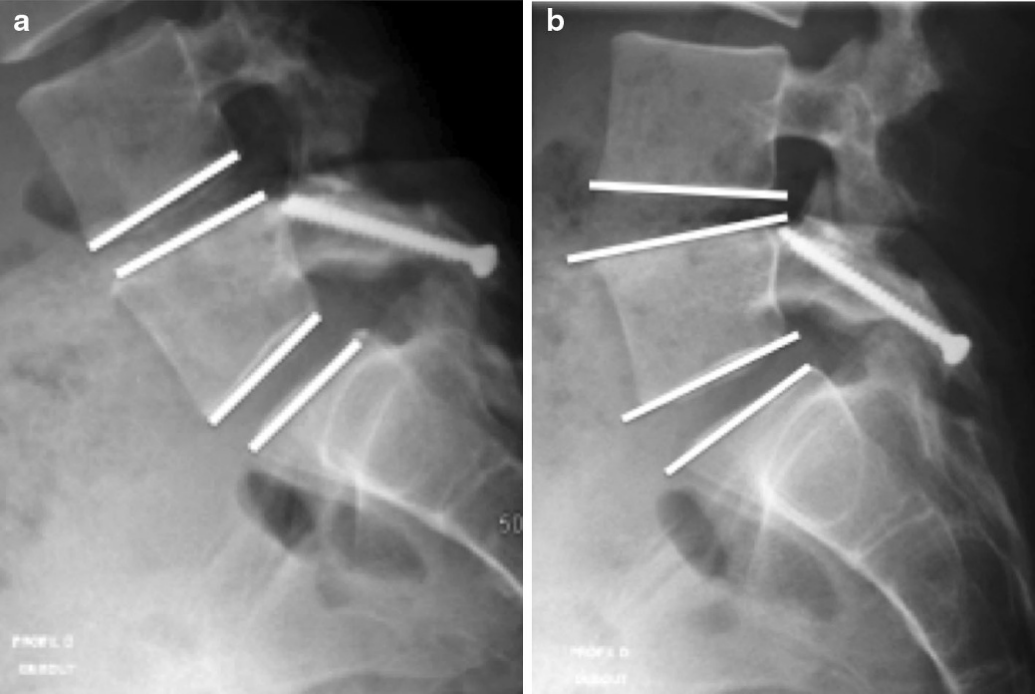
L4–L5 and L5–S1 motion measured in flexion (**a**) and in extension (**b**) according to Luk et al. [11]

Disk motion was calculated with the lateral lumbar spine in flexion and extension (Fig. 1). Motion at L4–L5 and L5–S1 were measured according to Luk et al. [11].

### Operative technique

**Fig. 2:**
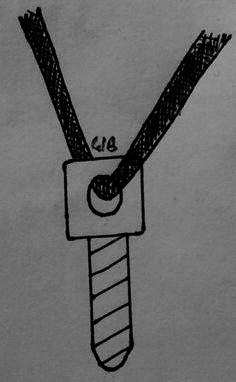
Screw with polyester ligament introduced in the head of the screw

**Fig. 3:**
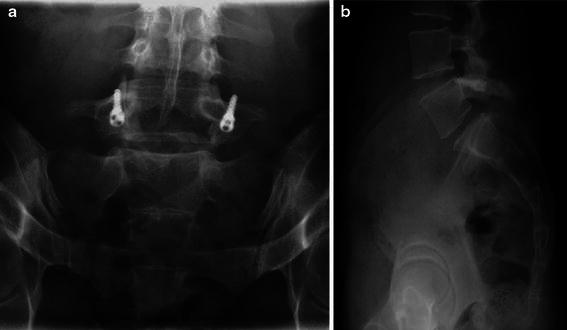
Position of the screw: diverging to about 5° and ascendant to about 20°

**Fig. 4:**
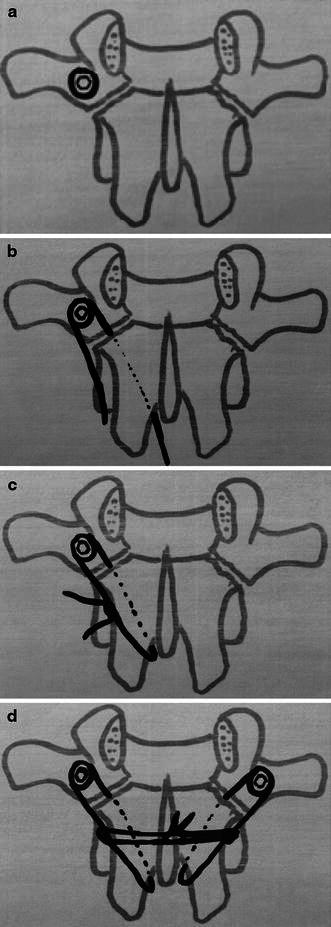
Positioning the transversal process screw (**a**); one strand of the ligament is passed below the lamina and the other above the lamina (**b**); the two strands are tied with a slip knot (**c**); the same procedure is done on the other side and both ligaments are tied under the spinous process (**d**)

The patient was positioned prone. A midline incision was made and the paraspinal musculature was elevated laterally to expose the lamina, the pars, and the base of the transverse process. Care was taken not to injure the capsule of the facet joint. The defect in the pars was exposed and the fibrocartilaginous elements curetted. A burr was used to decorticate the defect. The corresponding lamina and transverse process were exposed. For all patients, cancellous bone graft was harvested from the iliac posterior crest, placed in the defect, and impacted. For 20 patients (43.4 %) operated between 1988 and 2000, two pedicular screws (3.5-mm cortical screws) were passed obliquely across the defects, starting in the inferior margin of the lamina, as described by Buck [1]. Because of some technical difficulties due to hypoplasia of the posterior arch and the placement of the screw, this technique was modified. For 26 patients (56.6 %) operated between 2000 and 2010, lamina instrumentation was changed to a transversal process screw (Medicrea^®^) and a lamina polyester ligament (Fig. 2). On each side, a 4-mm screw was fixed to the transversal process after drilling. The screw was ascendant of about 20° and diverging of about 5° (Fig. 3). The length of the screw was standard: 20 mm. A polyester ligament was passed in the hole of the screwhead. The lamina was cleaned on both sides to pass the ligament. One strand of the ligament was passed below the lamina and the other under the lamina (Fig. 4). To pass the ligament below the lamina, we used prehensile pliers. Cancellous bone graft was placed on the pars defect before each ligament was tied on the lamina. The two ligaments were firmly fixed against the spinous process and the lamina, which promotes compression of the graft in the defect and stabilizes the posterior arch. Stabilization of the arch complex was confirmed by traction and the wound was closed with drainage.

Patients were allowed to stand up on the second or third postoperative day. Sitting position was authorized after surgery as soon as the patient was pain-free. A fiberglass jacket was applied before discharge home. This jacket was removed after 8 weeks. No physiotherapy was necessary after surgery.

### Statistical analysis

Student’s *t*-test was used for the statistical analysis of the motion L4–L5 and L5–S1 results. A *p*-value less than 0.05 was considered to indicate statistical significance.

## Results

Of the 46 operated patients, 35 (76 %; 17 female and 18 male) were included in the study and 11 patients were lost to follow-up. The mean age at the time of revision was 22.4 ± 6.3 years (range 13–40). Lysis was bilateral for all cases.

The clinical and radiological results were analyzed, with an average follow-up of 9.4 ± 5.52 years (range 2–24). At the final follow-up, the mean ODI was 12.6 ± 12.6 % (range 0–57.7). The clinical results of 31 patients were excellent (25 patients with ODI <20 %) or good (9 patients with 20 ≤ ODI < 39). One patient had severe disability (40 ≤ ODI < 59). It was not possible to compare before and after surgery because the preoperative ODI was unknown.

As measured on the flexion–extension radiographs, the mean mobility for L4–L5 was 11.8 ± 5.9° (range 0–22) and for the lumbo-sacral segment, it was 9.9 ± 5.7° (range 0–21). These results were compared to normal mobility values in adults according to Fairbank and Pynsent et al. [9]. The difference was not statistically significant (*p* > 0.05) for L4–L5 motion. The decrease was significant for the lumbo-sacral segment (*p* < 0.0005).

Five groups were identified based on the follow-up (Table 1). The mobility of each group was compared to the average of the group. No change of the segmental mobility over time was observed. except when the follow-up for L5–S1 was 5–10 years.

**Table 1:** L4–L5 and lumbo-sacral motion: no significant difference of mobility depending on follow-up

Follow-up	Number of patients	L4–L5 (°)	*p*-value	L5–S1 (°)	*p*-value
0–5 years	7	13.8 ± 6.23	>0.05	12.8 ± 5.84	>0.05
5–10 years	11	9 ± 5	>0.05	7.2 ± 2.7	=0.04
10–15 years	10	13 ± 6.5	>0.05	10.9 ± 7.4	>0.05
15–20 years	6	14.8 ± 5	>0.05	10.3 ± 6.2	>0.05
>20 years	1	5		7	

The fusion rate of the lysis was 91.4 % and pseudarthrosis was diagnosed in three patients:

- A symptomatic patient, first operated on before 2000, underwent a second operation 1 year later for pseudarthrosis. A bone graft was added for the left isthmus only and the left screw was changed.
- A second patient, operated in 2003, was pain-free for 6 years. Consolidation seemed sure on radiographs during this period. Mechanical pain appeared 6 years after surgery. Radiographs and CT confirmed recurrence. MRI showed disk degeneration. The patient was treated by postero-lateral fusion.
- A third patient, first operated on before 2000, was followed for 3 years after surgery without pain, and with consolidation on radiographs. At follow-up, she was asymptomatic (ODI = 0 %). Radiographs revealed recurrence of spondylolysis. Because the patient was asymptomatic, no surgery was proposed.

## Discussion

Direct repair of spondylolisthesis can save a functional segment in young patients. The aim of this study was to evaluate the long-term conservation of lumbo-sacral motion in patients following surgery involving a modification of Buck’s repair technique.

The advantages of direct pars repair by Buck’s technique include restoration of normal anatomy of the posterior elements, preservation of the functional motion segment in this group of young patients, less surgical trauma with the dissection limited to the medial side of the facet joint, less blood loss, and early functional recovery. However, careful patient selection is very important for the success of this procedure. The disk and facet joint at the involved level should be normal, without any signs of degeneration as assessed by MRI. For the last 5 years, before repair surgery, the state of the disk has been checked in our hospital. Almost all authors [12, 13] agree that degenerative abnormalities of the intervertebral disk or facet joints contraindicate direct repair surgery and prefer postero-lateral fusion.

Reconstruction of the pars interarticularis seems to be a logical and less aggressive approach for symptomatic patients, and reduces or relieves pain. Clinically, 97.1 % of patients in this group have excellent or good function outcomes according the ODI [9]. For lumbar surgical procedures, the minimum clinically significant difference in the ODI score has been calculated to be 12.8 points [14]. Westacott and Cooke [15] assessed the functional outcome after direct repair: the mean ODI was 8.9 but the follow-up was only 3.4 years (Table 2).

**Table 2:** Results of the functional Oswestry Disability Index (ODI) and follow-up in reported series of direct repair of the pars interarticularis

	Number of patients	Follow-up (years)	ODI (%)
Altaf et al. [16]	20	4	8
Debnath et al. [17]	22	2	10.7
Debnath et al. [18]	8	2	6.4
Koptan et al. [19]	10	4.5	11
Schlenzka et al. [20]	28	4.5	8.6
Our series	35	9.4	12.6

The most frequently used surgical procedure for symptomatic lumbar spondylolysis and spondylolisthesis is fusion [20]; this approach results in the loss of lumbo-sacral movement. Some investigators believe that the surgical fusion of the lumbar spine, and associated loss of motion at the fused site, may increase loading on the adjacent segments, and lead to symptomatic degeneration warranting additional surgery in 16.5 % of patents at 5 years and 36.1 % of patients at 10 years [7]. A surgical technique that allows anatomical repair of the isthmus and functional recovery of segmental motion would, therefore, be useful. White and Panjabi [21] have defined normal values in adults, as follows: 16° (range 9–21) for L4–L5 and 17° (range 10–24) for L5–S1. Values were compared to normal values in adults and to the results reported by Schlenzka et al. [22] for segmental fusion in Table 3. The mean flexion–extension mobility in the lowermost lumbar segments was lower than normal adult reference values. Segmental motion was also compared following fusion: there was no significant difference for L4–L5. The difference was highly significant for the lumbo-sacral segment (*p* < 0.0005). Roussouly et al. [23] demonstrated that populations with low-grade spondylolisthesis had less extension between L5 and S1 than in the normal population. We demonstrated that 9.9° is conserved in L5–S1.

**Table 3:** Comparison of the average motion of the lumbar spine after our technique or segmental fusion according to Schlenzka et al. [22] to normal values in adults according to White and Panjabi [21]

	Direct repair (°)	Segmental fusion (°) [22]	*p*-value	Normal (°) [21]
L4–L5	11.8 ± 5.9 (0–21)	15.2 ± 8 (0–30)	>0.05	16 (9–21)
L5–S1	9.9 ± 5.7 (0–21)	0.7 ± 2 (0–11)	<0.0005	17 (10–24)

Usually, the indications for direct isthmic repair are spondylolysis and spondylolisthesis with slight slipping in patients with painful symptoms resistant to conservative treatment and younger than 25 years of age [1].

Various procedures have been described for the direct repair of spondylolysis with consolidation (Table 4). Kimura [29] first described pars repair with isolated bone grafting of the defect, without internal fixation, and used postoperative casts for immobilization. Buck [1] first used a screw to stabilize the repair, in addition to bone grafting, and reported one failure in 16 patients. With this technique, problems can be encountered in seating the screwhead on the usually dysplastic lamina. This can cause fractures of the lamina, leading to loosening of the screw. Scott [30] used tension-band wiring and Morscher et al. [31] used a hook screw for the fixation of pars defects. Gillet and Petit [32] used a V-shaped rod–pedicle screw construct for pars fixation and reported satisfactory results in 70 % of the patients. This system using hook screws or V-shaped rod–pedicle constructs is sizeable. leading to conflict with the facet joint.

**Table 4:** Percentage of consolidation in reported series

Authors	Number of patients	Direct repair	Consolidation (%)
Pedersen and Hagen [5]	18	Buck	83
Bonnici et al. [24]	24	Buck	70
Pavlovcic [25]	17	Morscher	88
Altaf et al. [16]	20	Modular link	80
Fan et al. [8]	11	TSRH’s hook screw	100
Roca et al. [26]	15	Buck	87
Debnath et al. [17]	18	Scott/Buck	89
Albassir et al. [27]	37	Morscher	78
Kakiuchi [28]	16	Hook screw	100
Our series	35	Buck modified	91.4

Screw fixation has the biomechanical strength required to allow union of the defect. Between 1988 and 2000, two screws were passed obliquely across the defects, starting in the inferior margin of the lamina. as described by Buck [1]. Two patients (10 %) of the 20 operated using Buck’s technique [1] presented failure with pseudarthrosis. Lamina hypoplasia and difficulty in obtaining a good screw position made this procedure difficult. A modified Buck’s repair technique was created using ligament passed through the head of the screw. The screw is positioned on the base of the transversal process. Lamina is cleaned before passing the ligament. One strand of the ligament is passed above the lamina and the other under the posterior arch. Each ligament is tied on the lamina after the bone graft positioned on the defect. The two ligaments are then firmly fixed against the spinous process. No conflict occurs with the facet joint using this procedure. The success rate is consistent with those reported for other series, with consolidation in 91.4 % of cases.

Our modified Buck’s repair technique provides stiffness in interbody flexion–extension similar to that of the normal spine. This technique may be beneficial for maintaining motion at adjacent levels and for preventing shielding stress. Fan et al. [8] evaluated the biomechanical performance results of TSRH’s hook plus screw fixation after direct repair of spondylolysis defects in the pars interarticularis. They conclude that the intervertebral range of motion at L4–L5 is conserved, with approximately 10° in flexion–extension. Our results suggest that lumbo-sacral motion is still conserved 10 years after isthmic repair. This mobility prevents disk degenerative disease. It has been shown that lumbar fusion may deleteriously alter the sagittal balance of the spine [33, 34]. Intersegmental fusion alters the kinematics of the adjacent level, redistributing the mobility toward adjacent levels. The relative hypermobility at the adjacent levels may result in a significant acceleration of degenerative lesions at these sites, such that additional surgery may be required subsequently [35, 36].

This technique described in the present study seems to be a satisfying option in the treatment of patients with L5 spondylolisthesis with no associated degenerative disk disease. The success rate of consolidation is consistent with those reported in the literature. Using this technique, L4–L5 and L5–S1 motion is conserved in the long term. We think that preservation of lumbo-sacral mobility with this technique will prevent subsequent degenerative disk disease.
